# Cardiovascular risk assessment in periodontitis patients and controls using the European Systematic COronary Risk Evaluation (SCORE) model. A pilot study.

**DOI:** 10.3389/fphys.2022.1072215

**Published:** 2023-01-30

**Authors:** Madeline X. F. Kosho, Alexander R. E. Verhelst, Wijnand J. Teeuw, Victor E. A. Gerdes, Bruno G. Loos

**Affiliations:** ^1^ Department of Periodontology, Academic Centre for Dentistry Amsterdam (ACTA), University of Amsterdam and Vrije Universiteit Amsterdam, Amsterdam, Netherlands; ^2^ Department of Vascular Medicine, Amsterdam University Medical Center (AUMC), University of Amsterdam and Vrije Universiteit Amsterdam, Amsterdam, Netherlands; ^3^ Department of Internal Medicine, Spaarne Gasthuis, Hoofddorp, Netherlands

**Keywords:** periodontitis, periodontal disease, cardiovascular diseases, SCORE (systemic coronary risk evaluation), risk assessment, mortality

## Abstract

**Aim:** To investigate the use of the European SCORE model in a dental setting by exploring the frequency of a ‘high’ and ‘very high’ 10-year CVD mortality risk in patients with and without periodontitis. The secondary aim was to investigate the association of SCORE with various periodontitis parameters adjusting for remaining potential confounders.

**Material and methods:** In this study, we recruited periodontitis patients and non-periodontitis controls, all aged ≥40 years. We determined the 10-year CVD mortality risk per individual with the European Systematic Coronary Risk Evaluation (SCORE) model by using certain patient characteristics and biochemical analyses from blood by finger stick sampling.

**Results:** In total, 105 periodontitis patients (61 localized, 44 generalized stage III/IV) and 88 non-periodontitis controls were included (mean age: 54.4 years). The frequency of a ‘high’ and ‘very high’ 10-year CVD mortality risk was 43.8% in all periodontitis patients and 30.7% in controls (*p* = .061). In total, 29.5% generalized periodontitis patients had a ‘very high’ 10-year CVD mortality risk, compared to 16.4% in localized periodontitis patients and 9.1% in controls (*p* = .003). After adjustment for potential confounders, the total periodontitis group (OR 3.31; 95% CI 1.35–8.13), generalized periodontitis group (OR 5.32; 95% CI 1.90–14.90), lower number of teeth (OR .83; 95% CI .73–1.00) and higher number of teeth with radiographic bone loss ≥33% (OR 1.06; 95% CI 1.00–1.12) were associated with a “very high” SCORE category. In addition, various biochemical risk markers for CVD were more frequently elevated in periodontitis compared to controls (e.g., total cholesterol, triglycerides, C-reactive protein).

**Conclusion:** The periodontitis group as well as the control group had a sizable frequency of a ‘high’ and ‘very high’ 10-year CVD mortality risk. The presence and extent of periodontitis, lower number of teeth and higher number of teeth with bone loss ≥33% are significant risk indicators for a ‘very high’ 10-year CVD mortality risk. Therefore, SCORE in a dental setting can be a very useful tool to employ for primary and secondary prevention of CVD, especially among the dental attenders who have periodontitis.

## 1 Introduction

Cardiovascular disease (CVD) is a major global health burden. The amount of CVD deaths worldwide is continuously rising with an estimated number of 18.6 million deaths in 2019 (Roth et al., 2020). The rising CVD burden has led to the establishment of the European Alliance for Cardiovascular Health (EACH) to call for resolute action by the European Union (EU) (EACH, 2020). One of the main focuses of the EACH is to promote prevention through timely screening, early detection and diagnosis. However, systematic population-level screening for CVD risk and risk factors showed to have no effect on lowering CVD mortality and morbidity (Eriksen et al., 2021). Therefore, the European Heart Network (EHN), also part of the EACH, proposed to focus on more efficient targeted screening strategies in specific population groups, especially in those at risk for CVD (EHN, 2021). However, optimal strategies for CVD risk detection in selected at-risk populations are still being explored (EHN, 2021).

The United Kingdom National Health Service (NHS) also aims to reduce the CVD burden and is a step ahead in setting up a screening strategy. One of their plans is to detect CVD risk in a dental setting. One reason for choosing the dental setting is that patients tend to visit their dentist more regularly than their general physician (Crew, 2021). Also there is a favorable potential to detect CVD in a dental setting, because one of the comorbidities associated with CVD is periodontitis (Sanz et al., 2020a; b). Periodontitis is a chronic multifactorial inflammatory disease of the supporting structures of the teeth (root cementum, gingiva, periodontal ligament and alveolar bone) (Pihlstrom et al., 2005). It is highly conceivable that patients with periodontitis are more often at risk for CVD, since CVD and periodontitis share many risk factors, such as genetics (Aarabi et al., 2017), smoking (Tomar and Asma, 2000; Erhardt, 2009), overweight/obesity (Sowers, 2003; Suvan et al., 2011; Suvan et al., 2015), diabetes mellitus (DM) (Petrie et al., 2018; Sanz et al., 2018; Verhulst et al., 2019) and psychosocial stress (Genco et al., 1999; Steptoe and Kivimaki, 2012).

In the consensus report about “periodontitis and cardiovascular diseases” for oral health care professionals it was recommended to assess the CVD risk in periodontal patients and to inform patients about their CVD risk by using the European Systematic Coronary Risk Evaluation (SCORE) model (Piepoli et al., 2016; Sanz et al., 2020a; b). However, although advocated by the consensus, to date, there is no data available concerning CVD mortality risk assessment with SCORE in periodontitis patients in the dental setting. Also, within the dental setting in general, it is important to consider how many patients would fit into the category with a ‘high’ or ‘very high’ 10-year CVD mortality risk. These said individuals, if unaware of their condition, may need medical intervention or lifestyle advice to reduce the CVD risk.

Currently, limited studies report the CVD risk of patients attending general dental practices. Only one study, using SCORE in Swedish private dental offices, showed that the prevalence of an increased 10-year CVD mortality risk was 6% in a population of dental patients of ≥45 years (Jontell and Glick, 2009). Another study, using the comparable Framingham Risk score, showed that 17% of ≥40 years old dental patients in a university clinic, being unaware of their risk status, had an increased 10-year risk for CVD morbidity and mortality (Greenberg et al., 2007).

The aim of this pilot study is to investigate the use of the European SCORE model in a dental setting by exploring the frequency of a ‘high’ and ‘very high’ 10-year CVD mortality risk in patients with and without periodontitis. The secondary aim was to investigate the association of SCORE with various periodontitis parameters adjusting for remaining potential confounders. The findings of the current study will provide an indication illustrating the proportion of individuals in our dental population possibly in need of intervention to lower the CVD risk. Due to the rising CVD burden, we expect to see more patients with a ‘high’ or ‘very high’ 10-year CVD mortality risk in our dental population compared to the above-mentioned studies of Jontell and Glick 2009 and Greenberg et al., 2007; moreover, a vast amount of literature indicates that patients suffering from periodontal disease are more often at risk for CVD than dental attenders without periodontitis. Therefore, we hypothesize to observe in a periodontitis group a sizable number of individuals with a ‘high’ or ‘very high’ 10-year risk for CVD mortality.

## 2 Material and methods

### 2.1 Study design and patient recruitment

To investigate the use of the European SCORE model in a dental setting, the 10-year risk for CVD mortality was assessed in patients with and without periodontitis. Therefore, the following PECO question was formulated: Do patients attending the dental school clinic (P), with periodontitis (E) compared to those without periodontitis (C), show more often a ‘high’ and ‘very high’ 10-year risk for CVD mortality (O) applying SCORE?

In this study, we recruited patients referred to the Department of Periodontology of the Academic Centre for Dentistry of Amsterdam (ACTA) for diagnosis and treatment of periodontitis. Dental patients without periodontitis were consecutively recruited from the ACTA clinics for general dentistry, where appointments were scheduled for regular dental check-ups or restorative procedures. The enrolment period for all study participants was from March 2018 until March 2020. Recruitment was performed on patients with a minimum age of 40 years (minimum age to perform CVD risk assessment).

During the first referral visit, the patients underwent a full-mouth periodontal examination performed by periodontists or residents of the Department of Periodontology. We carried out measurements of probing pocket depth (PPD), gingival recessions and clinical attachment loss (CAL) for six sites per tooth using a manual probe. We also assessed molar furcation involvement and tooth mobility. We used recent dental radiographs (<1 year) to analyse interproximal alveolar bone levels.

Patients were initially screened for periodontitis according to the Centers for Disease Control and Prevention criteria–American Academy of Periodontology (CDC-AAP) case definition. They were asked to participate if positively diagnosed with periodontitis (≥2 interproximal sites with CAL ≥3 mm and ≥2 interproximal sites with PPD ≥4 mm, not on same tooth, or one site with PPD ≥5 mm) (Eke et al., 2012). Subsequently, we applied staging, grading and determination of the extent (localized or generalized) on each periodontitis case according to the consensus report criteria of the World Workshop on the classification of periodontal and peri-implant diseases and conditions (Papapanou et al., 2018). Control subjects took part if they failed to fulfil the criteria for the case definition of periodontitis, had not previously been treated for periodontitis, and did not have interproximal alveolar bone loss on recent bitewing radiographs (≤1 year old); a distance of ≤3 mm between the cemento-enamel junction to the most coronal part of the radiographic alveolar crest was accepted for a non-periodontitis, control subject.

All participants received verbal and written information about the purpose of the study and confirmed their consent. This study is part of a cross-sectional study: Periodontitis as signal for an underlying disease, registered at the ClinicalTrials.gov Identifier NCT03459638 and approved by the Medical Ethical Committee of the Amsterdam University Medical Center (2017.490 (A2019.151)-NL62337.029.17). The study was reported according to the STROBE-guidelines (Von Elm et al., 2007).

### 2.2 Clinical procedures

All participants received self-reported questionnaires to record age, sex, height, education degree (primary, secondary, >secondary [as proxy for socio-economic position]), smoking habits (none, former, current), presence of any CVD symptoms, cardiovascular event in the past (stroke, myocardial infarct), angioplasty/bypass in the past, hypertension, hypercholesterolemia, diabetes mellitus (DM), rheumatoid arthritis, potential CVD symptoms, first-degree relatives with CVD, first-degree relatives with DM, medication use and physical activity.

A clinical examination was performed to assess blood pressure, weight, waist- and hip circumference. After a sitting duration of at least 5 min in the dental chair, the blood pressure was measured three times on the right arm with a digital blood pressure monitor (Omron^®^, Hoofddorp, Netherlands). Blood pressure calculations were based on the average attained from the second and third measurement. Body weight, measured with a digital scale, and self-reported height were used to calculate the Body Mass Index (BMI). Waist and hip circumference were used to calculate the Waist-to-Hip Ratio (WHR). Waist circumference was recorded after exhaling at the approximate midpoint between the lower margin of the last palpable rib and the top of the iliac crest. Hip circumference was measured at the broadest part of the hip (WHO, 2011).

### 2.3 Blood collection and analysis of biochemical values

About 5–6 large drops of capillary blood using a finger stick was collected in a microtube containing 17 USP/ml lithium heparin. This method has been developed by a certified chemical laboratory (Labonovum B.V., Rotterdam, Netherlands) (Huijskens et al., 2019). The microtube was sent by normal mail to the chemical laboratory. The biochemical markers that were assessed were as follows; total cholesterol (elevated: ≥5 mmol/L), LDL-cholesterol (elevated: ≥3 mmol/L), HDL-cholesterol (lowered: ≤1 mmol/L), triglycerides (elevated: ≥2 mmol/L), CRP (elevated: >3 mg/L), HbA1c (elevated: ≥53 mmol/mol) and serum creatinine (Ridker, 2003; van ’t Riet et al., 2010; Nordestgaard et al., 2016). The eGFR (estimated glomerular filtration rate) was calculated using the CKD-EPI formula (Chronic Kidney Disease Epidemiology Collaboration) based on serum creatinine level, age, sex and race-ethnicity (Levey and Stevens, 2010). CKD was classified into 5 stages based on eGFR values in mL/min/1.73 m^2^: ≥90 (normal/high), 60–89 (mildly to moderately decreased), 30–59 (moderately to severely decreased), 15–29 (severely decreased) and <15 (kidney failure) (Levin et al., 2013). The blood test results were publicized on a secured digital platform provided by the laboratory. All patients also received the clinical values of their blood test. The measurements of total cholesterol and CRP were reported semiquantitively by the laboratory when these where below the cut-off value.

### 2.4 Cardiovascular risk assessment

The 10-year risk for CVD mortality was assessed according to SCORE, which is part of the European guidelines on cardiovascular disease prevention in clinical practice (Piepoli et al., 2016). To assess the CVD mortality risk in Europe, we distinguished between low and high-risk countries. Netherlands is a low-risk country. Therefore, to calculate CVD mortality risk, we applied the algorithm for a low-risk country. The SCORE algorithm is based on age, sex, smoking, systolic blood pressure, total cholesterol and HDL-cholesterol. The calculation was performed with the online version of SCORE: HeartScore (http://www.Heartscore.org), applicable to subjects ≥40 years up to 65. For subjects ≥65 years SCORE for older persons (SCORE O.P.) was used (Cooney et al., 2016).

SCORE has four risk classifications for a fatal CVD event within the next 10 years:

1. ‘Low’ risk (calculated SCORE <1%)
2. ‘Moderate’ risk (calculated SCORE ≥1 and <5%)
3. ‘High’ risk (calculated SCORE ≥5 and <10%)
4. ‘Very high’ risk (calculated SCORE ≥10%)

In addition, SCORE classifies patients directly in the ‘high’ risk category when at least one of the following characteristics is present: Total cholesterol >8 mmol/L, systolic blood pressure ≥180 mmHg and/or diastolic blood pressure ≥110 mmHg, presence of DM (self-reported or HbA1c ≥53 mmol/mol) or mild to moderate CKD (GFR 30–59 mL/min/1.73 m^2^). Patients with the following characteristics were directly classified into the ‘very high’ risk category: Documented CVD event, severe CKD (GFR <30 mL/min/1.73 m^2^) or DM combined with a target organ damage (e.g. proteinuria) or combined with a major risk factor (smoking, hypercholesterolemia, hypertension) (Piepoli et al., 2016).

For subjects without self-reported DM, the HbA1c cut-off for the suspected presence of DM was defined at ≥53 mmol/mol (≥7%). This threshold had a specificity of 100% in the Dutch population, excluding possible false positive measurements (van ’t Riet et al., 2010).

### 2.5 Data analysis

No power calculation was performed for the current research question. Therefore, the results are considered preliminary and explorative. Data was analysed with SPSS 25.9.6.0.0 (IBM SPSS, Chicago, IL, United States). Means, standard deviations, range and frequency distributions were calculated. Missing data for some biochemical parameters have been excluded. Background variables, demographic data and biochemical measurements were compared with independent samples *t*-test or by chi-squared tests. ANOVA or chi-squared tests (linear by linear) were used when comparing three groups (non-periodontitis controls, patients with localized periodontitis, patients with generalized periodontitis). A binary logistic regression analysis was performed to evaluate the association between the presence and extent of periodontitis, periodontal parameters and SCORE. Independent variables were adjusted for potential confounders that were not included in the calculation for SCORE (education, physical activity, abdominal obesity and first-degree relative with CVD). Odds ratios (OR) and 95% confidence intervals (95% CI) were calculated. The significance level was set at *p* < .05.

## 3 Results

### 3.1 Background characteristics

**FIGURE 1:**
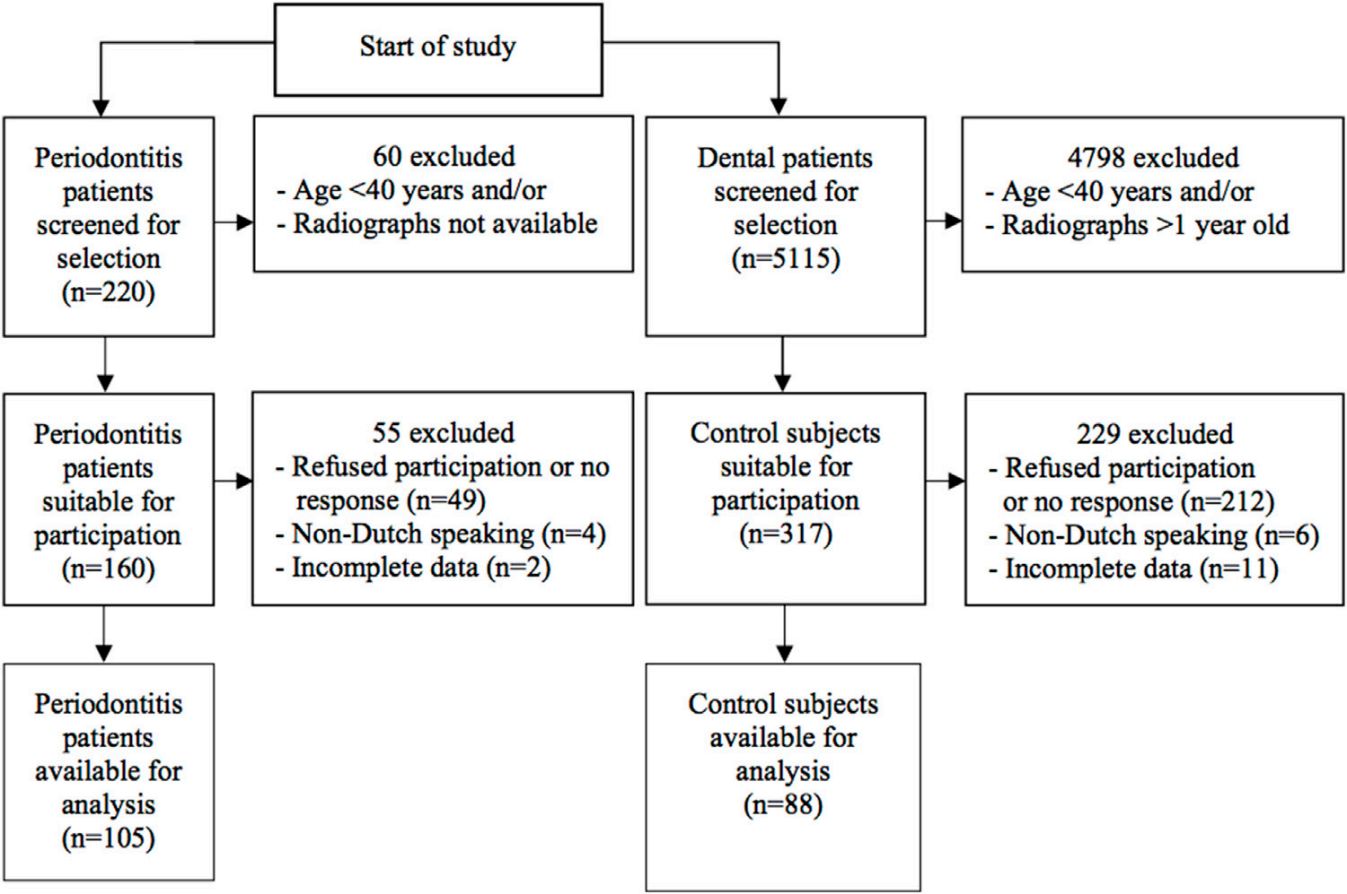
Flowchart of inclusion of periodontitis patients and control subjects.

We included a total of 88 non-periodontitis controls and 105 periodontitis patients in this study (Figure 1). Demographic characteristics are shown in Table 1. The mean age for control subjects was 54.8 years and for periodontitis patients 53.9 years (*p =* .515). There was no significant difference between the age categories in the control and periodontitis group (*p* = .991). A higher percentage of control subjects (60.2%) were educated beyond secondary education level compared to periodontitis patients (42.9%) (*p* = .045). Among periodontitis patients, there were more current smokers (38.1%) than among controls (10.2%) (*p* < .001).

**TABLE 1:** Demographic characteristics of the study population.

	Control (n = 88)	Periodontitis (n = 105)	*p*-value
Age (years)	54.8 ± 9.6	53.9 ± 8.8	.515
Age categories			.991
40–49	28 (31.8)	34 (32.4)	
50–59	34 (38.6)	42 (40.0)	
60–64	13 (14.8)	15 (14.3)	
≥65	13 (14.8)	14 (13.3)	
Sex			.724
Male	50 (56.8)	57 (54.3)	
Female	38 (43.2)	48 (45.7)	
Education^a^			.045
Primary	11 (12.5)	23 (21.9)	
Secondary	24 (27.3)	37 (35.2)	
Beyond Secondary	53 (60.2)	45 (42.9)	
Smoking status			<.001
Current	9 (10.2)	40 (38.1)	
Former	29 (33.0)	36 (34.3)	
Never	50 (56.8)	29 (27.6)	

*Note*. Data are presented as the mean ± SD or as n (%).

^a^ Primary: primary education or preparatory secondary vocational education. Secondary: higher secondary general education, pre-university education.

Dental and periodontal parameters are shown in Table 2. Significantly less teeth were present in periodontitis patients (25.7) than in controls (27.4) (*p* = .012). All periodontitis patients were categorized into stage III (73.3%) or stage IV (26.7%). There were 58.1% localized and 41.9% generalized periodontitis cases. All periodontitis cases were classified as grade B (32.4%) or grade C (67.7%).

**TABLE 2:** Dental and periodontal parameters.

	Control (n = 88)	Periodontitis (n = 105)	*p*-value
Number of teeth	27.4 ± 2.2	25.7 ± 3.0	.012
Number of teeth with ≥33% bone loss	NA	8.7 ± 6.7	NA
Number of teeth with PPD ≥6 mm	NA	8.6 ± 6.3	NA
Number of sites with PPD ≥6 mm	NA	18.7 ± 18.9	NA
Severity^a^			NA
Stage III	NA	77 (73.3)	
Stage IV	NA	28 (26.7)	
Extent^a^			NA
Localized	NA	61 (58.1)	
Generalized	NA	44 (41.9)	
Grade^a^			NA
A	NA	0 (.0)	
B	NA	34 (32.4)	
C	NA	71 (67.7)	

*Note*. Data are presented as the mean ± SD or as *n* (%). NA, not applicable; PPD, probing pocket depth.

^a^ Staging, extent and grading according to World Workshop 2017 (Papapanou et al., 2018).

Based on the fact that the inflammatory burden in generalized periodontitis is larger than in localized periodontitis, we applied this distinction in the group with periodontitis patients. The periodontitis group consisted of 61 patients with periodontitis localized stage III/IV and 44 patients with periodontitis generalized stage III/IV. Table 3 shows the self-reported CVD parameters within the three groups (control, localized and generalized periodontitis) and also for the total periodontitis group. More patients with generalized periodontitis (12.2%) reported a myocardial infarct in the past compared to localized periodontitis patients (.0%) and controls (1.1%) (*p* = .004). The number of days per week with physical activity of ≥30 min/day was lower in localized (3.9) and generalized periodontitis patients (3.6) compared to controls (4.8) (*p* = .027). The total periodontitis group reported more often the presence of rheumatoid arthritis (7.6%) compared to the control group (.0%) (*p* = .008). Periodontitis patients also showed a significantly higher frequency of fatigue after exercise (23.5%) compared to the controls (10.2%) (*p* = .016).

**TABLE 3:** Data from patient’s health history (self-reported).

	Control (n = 88)	Localized Periodontitis (n = 61)	Generalized Periodontitis (n = 44)	*p*-value^a^	Total Periodontitis (n = 105)	*p*-value^b^
Presence of CVD	7 (8.0)	8 (13.1)	8 (18.2)	.082	16 (15.2)	.120
Stroke	1 (1.1)	3 (4.9)	2 (4.9)	.191	5 (4.9)	.139
Myocardial infarct	1 (1.1)	0 (.0)	5 (12.2)	.004	5 (4.9)	.139
Angioplasty/bypass	1 (1.1)	2 (3.3)	4 (9.8)	.021	6 (5.9)	.083
Hypertension	21 (23.9)	14 (23.0)	14 (31.8)	.387	28 (26.7)	.656
Hypercholesterolemia	15 (17.0)	17 (27.9)	10 (22.7)	.317	27 (25.7)	.146
Diabetes mellitus	3 (3.4)	6 (9.8)	4 (9.1)	.153	10 (9.5)	.091
Rheumatoid arthritis	0 (.0)	6 (9.8)	2 (4.5)	.083	8 (7.6)	.008
Potential CVD symptoms						
Chest pain or discomfort	4 (4.5)	6 (9.8)	2 (4.9)	.714	8 (7.8)	.351
Shortness of breath	15 (17.0)	11 (18.0)	6 (14.6)	.790	17 (16.7)	.945
Fatigue after exercise	9 (10.2)	18 (29.5)	6 (14.6)	.210	24 (23.5)	.016
Heart palpitations	7 (8.0)	11 (18.0)	5 (12.2)	.298	16 (15.7)	.103
Swollen ankles	10 (11.4)	10 (16.4)	2 (4.9)	.462	12 (11.8)	.931
Pain in the legs after walking	6 (6.8)	12 (19.7)	0 (.0)	.627	12 (11.8)	.246
Sudden (temporary) loss of speech, vision, strength or sensation	9 (10.2)	6 (9.8)	1 (2.4)	.180	7 (6.9)	.405
First-degree relative with CVD	45 (51.1)	27 (44.3)	26 (60.5)	.471	53 (51.0)	.981
First-degree relative with diabetes mellitus	31 (35.2)	29 (47.5)	13 (29.5)	.987	42 (40.0)	.496
Medication use for CVD	15 (15.9)	15 (24.6)	11 (25.0)	.173	26 (24.8)	.131
Anticoagulants/antiplatelets	5 (5.7)	6 (9.8)	3 (6.8)	.678	9 (8.6)	.441
Beta-blockers	2 (2.3)	3 (4.9)	4 (9.1)	.083	7 (6.7)	.149
ACE inhibitors	8 (9.1)	9 (14.8)	6 (13.6)	.366	15 (14.3)	.267
Calcium antagonists	6 (6.8)	5 (8.2)	2 (4.5)	.709	7 (6.7)	.967
Diuretics	2 (2.3)	4 (6.6)	2 (4.5)	.409	6 (5.7)	.232
Statins	6 (6.8)	8 (13.1)	6 (13.6)	.177	14 (13.3)	.139
Nitrates	0 (.0)	0 (.0)	1 (2.3)	.122	1 (1.0)	.359
Physical activity ≥3 min (days/week)	4.8 ± 2.4	3.9 ± 2.4	3.6 ± 2.8	.027	3.8 ± 2.5	.009

*Note*. Data are presented as the mean ± SD or as *n* (%). CVD, cardiovascular disease.

^a^ Overall *p*-value for control, localized periodontitis and generalized periodontitis.

^b^ Overall *p*-value for control and total periodontitis.

Clinical parameters related to general health and CVD are shown in Table 4. No significant difference between the total periodontitis group compared to the control group was found for hypertension based on blood pressure measurements (*p* = .304), BMI (*p* = .077) or BMI categories (*p* = .183). Periodontitis patients showed a significant higher mean waist circumference (*p* = .010) and a higher mean Waist-to-Hip Ratio (WHR) (*p* = .004) compared to the controls. Also, there was a significant difference for the proportion of patients with abdominal obesity among controls (60.2%), localized (68.9%) and generalized (80.5%) periodontitis (*p* = .022).

**TABLE 4:** Results of clinical assessments of parameters related to general health and cardiovascular disease.

	Control(n = 88)	Localized Periodontitis(n = 61)	Generalized Periodontitis(n = 44)	*p*-value^d^	TotalPeriodontitis(n = 105)	*p*-value^e^
SBP (mmHg)	130 ± 20.3	132 ± 8.0	134 ± 15.7	.546	133 ± 17.1	.347
≥140	30 (34.1)	14 (23.0)	10 (22.7)	.122	24 (22.9)	.083
≥180	1 (1.1)	2 (3.3)	0 (.0)	.818	2 (1.9)	.667
DBP (mmHg)	83 ± 11.9	83 ± 9.9	84 ± 9.5	.845	83 ± 9.7	.646
≥90	23 (26.1)	10 (16.4)	10 (22.7)	.488	20 (19.0)	.239
≥110	3 (3.4)	1 (1.6)	0 (.0)	.186	1 (1.0)	.233
Hypertension^a^	33 (37.5)	17 (27.9)	15 (34.1)	.543	32 (30.5)	.304
BMI (kg/m^2^)	26.0 ± 4.6	26.9 ± 4.5	27.4 ± 4.1	.186	27.1 ± 2.0	.077
BMI category (kg/m^2^)				.061		.183
<25	41 (46.6)	26 (42.6)	13 (29.5)		39 (37.1)	
≥25 and <30^b^	34 (38.6)	19 (31.1)	21 (47.7)		40 (38.1)	
≥30^b^	13 (14.8)	16 (26.2)	10 (22.7)		26 (24.8)	
Waist circumference (cm)	92.0 ± 15.1	96.3 ± 12.2	98.5 ± 12.9	.025	97.2 5 ± 12.5	.010
Waist-to-Hip Ratio	.88 ± .12	.91 ± .07	.94 ± .09	.006	.92 ± .08	.004
Abdominal obesity^c^	53 (60.2)	42 (68.9)	33 (80.5)	.022	75 (73.5)	.051

*Note*. Data are presented as the mean ± SD or as *n* (%). SBP/DBP, Systolic/Diastolic Blood Pressure; BMI, Body Mass Index; WHR, Waist-to-Hip Ratio.

^a^ Measurement of ≥140 mmHg systolic and/or ≥90 mmHg diastolic (average of two measurements).

^b^ BMI determined overweight (≥25) and obesity (≥30).

^c^ WHR determined Abdominal obesity, if WHR ♀ >.85, ♂ >.90 (World Health Organization, 2011).

^d^ Overall *p*-value for control, localized periodontitis and generalized periodontitis.

^e^ Overall *p*-value for control and total periodontitis.

### 3.2 Biochemical values obtained with finger stick sampling procedure

Results of measured biochemical values are shown in Table 5. A higher frequency of patients in the total periodontitis group (34.6%) had an elevated level of total cholesterol (≥5 mmol/L) compared to the control group (18.4%) (*p* = .012). There was a significant difference in the proportion of patients with an elevated level of LDL-cholesterol (≥3 mmol/L) between the control group (23.0%), localized (32.8%) and generalized periodontitis (39.5%) (*p* = .044). We saw the same trend for the proportion of patients with an elevated level of triglycerides (≥2 mmol/L) in the control group (25.3%), localized (37.7%) and generalized periodontitis group (44.2%) (*p* = .023). Also, this trend was seen for the number of patients with an elevated CRP level (>3 mg/L) among the control group (7.0%), localized (21.3%) and generalized (27.5%) periodontitis patients (*p* = .002). The difference in the frequency of subjects with an elevated HbA1c level (≥53 mmol/mol) between the three groups was not significant (*p* = .094). Also, we saw no differences between the three groups for HDL-cholesterol (*p* = .246) and serum creatinine (*p* = .356). The eGFR was the highest among the patients with generalized periodontitis (89.1 mL/min/1.73 m^2^) compared to controls (79.6 mL/min/1.73 m^2^) and localized periodontitis (77.3 ml/min/1.73 m^2^) (*p* = .044).

**TABLE 5:** Results of measured biochemical values.

	Control (n = 88)	Localized Periodontitis (n = 61)	Generalized Periodontitis (n = 44)	*p*-value^f^	Total Periodontitis (n = 105)	*p*-value^g^
Total cholesterol ≥5 (mmol/L)^a^	16 (18.4)	21 (34.4)	15 (34.9)	.025	36 (34.6)	.012
Total cholesterol >8 (mmol/L)	1 (1.1)	1 (1.6)	1 (2.3)	.618	2 (1.9)	.667
LDL-cholesterol (mmol/L)^b^	2.4 ± 1.0	2.7 ± 1.1	2.8 ± 0.9	.022	2.8 ± 1.0	.006
LDL-cholesterol ≥3 (mmol/L)	20 (23.0)	20 (32.8)	17 (39.5)	.044	37 (35.6)	.058
HDL-cholesterol (mmol/L)^b^	1.10 ± 0.5	1.22 ± 0.5	1.18 ± 0.4	.246	1.21 ± 0.5	.105
HDL-cholesterol ≤1 (mmol/L)	39 (44.8)	22 (36.1)	17 (39.5)	.455	39 (37.5)	.305
Triglycerides (mmol/L)^b^	1.81 ± 1.0	2.03 ± 1.2	2.06 ± 1.4	.404	2.04 ± 1.3	.179
Triglycerides ≥2 (mmol/L)	22 (25.3)	23 (37.7)	19 (44.2)	.023	42 (40.4)	.028
CRP >3 (mg/L)^c^	6 (7.0)	13 (21.3)	11 (27.5)	.002	24 (23.8)	.002
HbA1c ≥53 (mmol/mol)^d^	9 (10.2)	11 (18.3)	9 (20.5)	.094	20 (19.2)	.083
Serum creatinine (µmol/L)^e^	87.4 ± 23.5	93.7 ± 53.9	83.2 ± 26.5	.356	89.5 ± 45.2	.701
eGFR (mL/min/1.73 m^2^)^e^	79.6 ± 21.0	77.3 ± 22.9	89.1 ± 29.0	.044	82.0 ± 26.0	.499
eGFR (mL/min/1.73 m^2^)^e^				.216		.291
G1 ≥90	27 (31.4)	24 (40.7)	18 (46.2)		42 (42.9)	
G2 60–89	45 (52.3)	22 (37.3)	17 (43.6)		39 (39.8)	
G3 30–59	14 (16.3)	11 (18.6)	4 (10.3)		15 (15.3)	
G4 15–29	0 (.0)	1 (1.7)	0 (.0)		1 (1.0)	
G5 <15	0 (.0)	1 (1.7)	0 (.0)		1 (1.0)	

*Note.* Data are presented as the mean ± SD or as n (%). LDL, low density lipoprotein; HDL, high density lipoprotein; eGFR, estimated Glomerular Filtration Rate; HbA1c, Glycated haemoglobin; CRP, C-reactive Protein.

Missing values for:

^a^ Control: n = 1.

^b^ Control: n = 1, generalized periodontitis: n = 1.

^c^ Control: n = 2, generalized periodontitis: n = 4.

^d^ Localized periodontitis: n = 1.

^e^ Control: n = 1, localized periodontitis: n = 2; generalized periodontitis: n = 5.

^f^ Overall *p*-value for control, localized periodontitis and generalized periodontitis.

^g^ Overall *p*-value for control and total periodontitis.

### 3.3 Cardiovascular risk assessment

**FIGURE 2:**
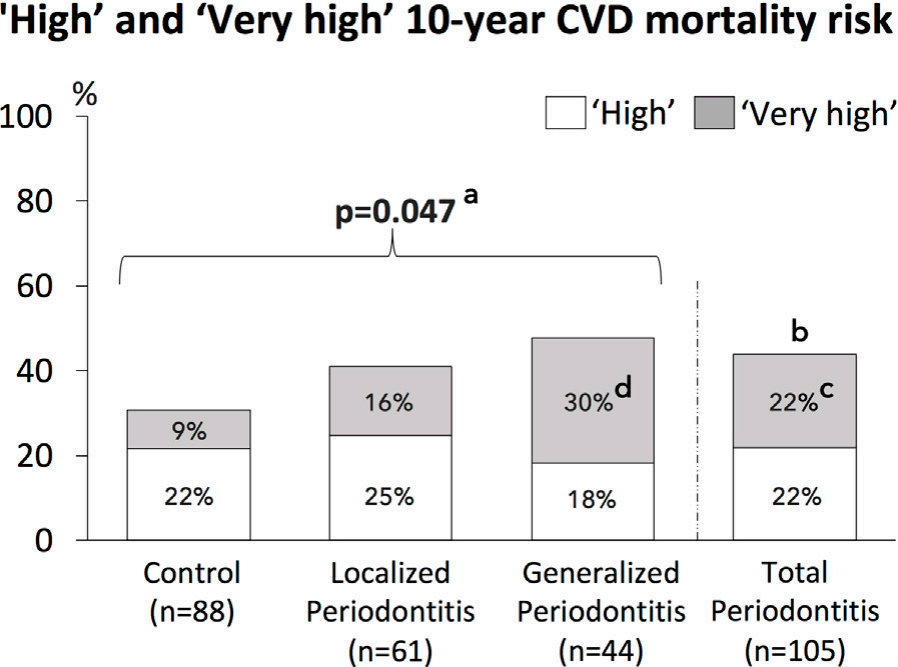
Frequencies of ‘High’ and ‘Very high’ 10-year CVD mortality risk for the study population, consisting of controls, patients with localized periodontitis and generalized periodontitis, as well as all periodontitis patients combined (total periodontitis).**a** = The overall *p*-value for the difference of combined ‘high’ and ‘very high’ 10-year CVD mortality risk between the 3 groups (control, localized and generalized periodontitis). **b** = *p*=0.061 for the difference of combined ‘high’ and ‘very high’ 10-year CVD mortality risk between the total periodontitis group and control group. **c** = The ‘very high’ 10-year CVD mortality risk was significantly increased in the total periodontitis group compared to the control group (*p*=0.016).**d** = The overall *p*-value for the difference of ‘very high’ 10-year CVD mortality risk between the 3 groups (control, localized and generalized periodontitis) was *p*=0.003. Post-hoc testing showed that the ‘very high’ 10-year CVD mortality risk was significantly increased in the group with generalized periodontitis compared to the control group (*p*=0.015, after correction for multiple testing).

The frequency of the four SCORE risk categories (‘low’, ‘moderate’, ‘high’, ‘very high’) was significantly different among the three groups (control, localized and generalized periodontitis) (*p* = .029) and among the control group and total periodontitis group (*p* = .034) (Table 6). Figure 2 shows the proportion of patients with combined “high” and “very high” 10-year risk for CVD mortality, which was the highest among patients with generalized periodontitis (47.7%) and localized periodontitis (41.0%), when compared to controls (30.7%) (*p* = .047). The frequency of a ‘high’ and ‘very high’ 10-year CVD mortality risk was 43.8% in the total periodontitis group and 30.7% in the control group (*p* = .061). The proportion of patients with a ‘very high’ 10-year risk for CVD mortality was the highest among patients with generalized periodontitis (29.5%), compared to localized periodontitis (16.4%) and controls (9.1%) (*p* = .003). The proportion of patients with a ‘very high’ 10-year CVD mortality risk was significantly increased in the total periodontitis group compared to the control group (*p* = .016) (Figure 2).

**TABLE 6:** Cardiovascular risk assessment with SCORE: 10-year risk on CVD mortality.

SCORE classification	Control (n = 88)	Localized Periodontitis (n = 61)	Generalized Periodontitis (n = 44)	p-value^a^	Total Periodontitis (n = 105)	p-value^b^
				.029		.034
“Low”	12 (13.6)	14 (23.0)	5 (11.4)		19 (18.1)	
“Moderate”	49 (55.7)	22 (36.1)	18 (40.9)		40 (38.1)	
“High”	19 (21.6)	15 (24.6)	8 (18.2)		23 (21.9)	
“Very high”	8 (9.1)	10 (16.4)	13 (29.5)		23 (21.9)	

*Note*. Data are presented as n (%). SCORE, Systematic Coronary Risk Evaluation (www.heartscore.org); CVD, cardiovascular disease.

^a^ Overall *p*-value for control, localized periodontitis and generalized periodontitis.

^b^ Overall *p*-value for control and total periodontitis.

Generalized periodontitis (OR 4.19; 95% CI 1.58–11.10) and total periodontitis (OR 2.81; 95% CI 1.19–6.64) were significantly associated with a ‘very high’ 10-year CVD mortality risk (Table 7A). After adjustment for education, physical activity, abdominal obesity and first-degree relative with CVD, generalized periodontitis (OR 5.32; 95% CI 1.90–14.90) and total periodontitis (OR 3.31; 95% CI 1.35–8.13) remained significantly associated with the ‘very high’ risk SCORE category. In addition, generalized periodontitis also showed a significant association with the combined ‘high’ and ‘very high’ (OR 2.30; 95% CI 1.04–5.06) SCORE category (Table 7A).

**TABLE 7:** Associations between the extent of periodontitis (Part A), clinical periodontal parameters (Part B) and SCORE.

A						
SCORE classification	Localized periodontitis		Generalized periodontitis		Total periodontitis	
	Crude OR	Adjusted OR*	Crude OR	Adjusted OR*	Crude OR	Adjusted OR*
“High” and “Very high”	1.57 (.79−3.10)	1.54 (.76-3.13)	2.06 (.98−4.35)	2.30^a^ (1.04−5.06)	1.76 (.97−3.19)	1.81 (.97−3.38)
“Very high”	1.96 (.73−5.30)	2.24 (.80−6.26)	4.19^b^ (1.58−11.10)	5.32^c^ (1.90−14.90)	2.81^d^ (1.19−6.64)	3.31^e^ (1.35−8.13)

B						
SCORE classification	Number of teeth		Number of teeth with bone loss ≥33%		Number of teeth with PPD ≥6 mm	
	Crude OR	Adjusted OR*	Crude OR	Adjusted OR*	Crude OR	Adjusted OR*
“High” and “Very high”	.90^f^ (.81−1.00)	.88^g^ (.78−.98)	1.03 (.98−107)	1.03 (.99−1.08)	1.01 (.96−1.06)	1.02 (.97−1.07)
“Very high”	.87^h^ (.77−1.00)	.83^i^ (.72−1.00)	1.05 (1.00−1.11)	1.06^j^ (1.00−1.12)	1.03 (.97−1.09)	1.03 (.97−1.10)

Part A. Values represent crude and adjusted odds ratio’s (OR) and 95% confidence intervals (in parenthesis) for localized periodontitis vs. control, generalized periodontitis vs. control and total periodontitis vs. control. * Adjusted for education, physical activity, abdominal obesity, first-degree relative with CVD.

^a^ p = .039.

^b^ p = .004.

^c^ p = .001.

^d^ p = .019.

^e^ p = .009.

Part B. Values represent crude and adjusted odds ratio’s (OR) and 95% confidence intervals (in parenthesis) for the number of teeth, number of teeth with radiographic bone loss of ≥33% and number of teeth with periodontal pocket depth (PPD) ≥6 mm. * Adjusted for education, physical activity, abdominal obesity, first-degree relative with CVD.

^f^ p = .040.

^g^ p = .022.

^h^ p = .042.

^i^ p = .013.

^j^ p = .035.

There was a significant negative association between the number of teeth and the combined ‘high’ and ‘very high’ (OR .90; 95% CI .81–1.00) and ‘very high’ (OR .87; 95% CI .77–1.00) SCORE categories, which remained significant after adjustment for potential confounders (OR .88; 95% CI .78–.98; OR .83; 95% CI .72–1.00, respectively). The number of teeth with radiographic bone loss ≥33% was significantly associated with the ‘very high’ SCORE category after adjustment for confounders (OR 1.06; 95% CI 1.00–1.12). No significant associations were observed between the number of teeth with PPD ≥6 mm and SCORE (Table 7B).

## 4 Discussion

In this pilot study, we investigated the use of the European SCORE model in a dental setting to explore the frequency of a ‘high’ and ‘very high’ 10-year CVD mortality risk in patients with and without periodontitis. We also studied the association of various periodontitis parameters with a ‘high’ and ‘very high’ 10-year CVD mortality risk. The frequency of the combined ‘high’ and ‘very high’ 10-year CVD mortality risk was 43.8% in the periodontitis group and 30.7% in the control group. When we considered a ‘very high’ 10-year CVD risk in relation to a localized and generalized form of periodontitis, then this study showed that the frequency for a ‘very high’ 10-year CVD mortality risk was 9.1% for the control group, 16.4% for the localized periodontitis group and 29.5% for the generalized periodontitis group, indicating that the extent of periodontitis also plays a role. In our dental school, the control group as well as the periodontitis group had a sizable frequency of a ‘high’ and ‘very high’ 10-year CVD mortality risk. The presence of periodontitis, the extent of periodontitis, as well as a lower number of teeth and a higher number of teeth with bone loss ≥33% are risk indicators for a ‘very high’ 10-year CVD mortality risk. Therefore, the conclusion of the current study is that applying SCORE in a dental setting can be a very useful tool for primary and secondary prevention of CVD, especially among the dental attenders who have periodontitis.

This study has a number of strengths - including 1) using the most recent classification of periodontal diseases (Papapanou et al., 2018); 2) using the European SCORE system for CVD mortality risk assessment (Piepoli et al., 2016) and also recommended by consensus reports (Sanz et al., 2020a; b); 3) using a novel blood sampling device (finger stick), which is a clinically validated alternative for venepuncture sampling and applicable in a dental setting (Huisman, 2017; Huijskens et al., 2019). A limitation is that the current research is a pilot study and no *a priori* power calculation has been performed. The study needs replication, and we expect that the current results are useful to design a full-powered study. The results of this study are based on a dental school patient population, consisting of individuals living in Amsterdam and surrounding area. To confirm our findings, future studies should perform a CVD risk assessment in periodontal offices in other regions and other European countries. Of note, in the process of this pilot study, we found that obtaining sufficient drops of blood with a finger stick required a small learning curve; initially, in some instances, we failed to collect the required amount of blood.

Although the European SCORE system has been recently updated by SCORE2 (ESC, 2021), for the current study, SCORE was used (Piepoli et al., 2016). One of the differences of SCORE2 with SCORE is that SCORE2 is not applicable for patients with DM, because they are generally considered at high risk for CVD. Interestingly in the current study, the number of patients with suspected diagnosis of DM (measured HbA1c ≥53 mmol/mol) was higher than the number of patients with self-reported DM; in a previous study from our group, we reported “undiagnosed diabetes” for 18% in a dental school patient population with severe periodontitis (Teeuw et al., 2017). By using SCORE, it was possible to include these patients as well, and therefore being more successful for primary and secondary CVD prevention. Another advantage of SCORE is that patients with self-reported DM can be distinguished belonging to the 10-year ‘high’ or ‘very high’ CVD mortality risk based on the concomitant presence of one other major risk factor next to DM (smoking, hypercholesterolemia, hypertension). Furthermore, a recent study has compared the performance of 22 risk prediction scores for CVD in patients with DM type 2 (Dziopa et al., 2022). From all models, SCORE performed best for predicting CVD. In this way, SCORE based on clinical characteristics and blood biochemistry analysis, is the best method suitable in a dental practice environment for CVD risk screening.

The results of our study show the clear increased CVD risk with an increasing extent of periodontitis, applying the classification criteria of the World Workshop 2017 (Papapanou et al., 2018). Confirmatory to previous studies, we also observed that biochemical risk markers for CVD, such as elevated total cholesterol, LDL-cholesterol and triglycerides, showed an increasing trend in localized and generalized periodontitis patients compared to controls (Losche et al., 2000; Nibali et al., 2007; Monteiro et al., 2009; Schenkein and Loos, 2013). The above findings clearly confirm the importance of the extent of the periodontitis-related inflammatory burden; as the extent of periodontally-involved teeth increases, the extent of the inflammatory burden increases and this contributes to systemic inflammation (Schenkein and Loos, 2013; Sanz et al., 2020a; b). Similarly, we also noted the extent-related increasing frequency of individuals with CRP >3.0 mg/L from control to localized periodontitis to generalized periodontitis, which also has been previously reported (Loos et al., 2000). According to the American Heart Association and Centers for Disease Control and Prevention, subjects with CRP levels >3.0 mg/L are considered to have a ‘high’ risk for a CVD event (Pearson et al., 2003; Yang et al., 2021).

In total 69 (36%) of 193 study participants had a ‘high’ or ‘very high’ 10-year CVD mortality risk in the current study population. From these 69 individuals with an increased CVD risk, 37% with periodontitis and 17% of the controls were already aware that they suffered from hypertension, DM or CVD based on their self-reported health history. However, although these patients were apparently diagnosed and presumably treated for these conditions, several of them still had physical and biochemical measurements above the normal range for one or more of these items: elevated blood pressure, elevated LDL (≥2.6 mmol/mol), elevated HbA1c (≥53 mmol/mol). In these cases, it is possible that patients do not receive the optimal dose for their medication or did not visit their physician/specialist recently for check-ups. Therefore, for patients already self-reporting aspects of CVD, it is still useful to perform a CVD risk assessment to contribute to secondary prevention.

Considering the results of the present study, demonstrating a moderate frequency of a ‘high’ and ‘very high’ 10-year CVD mortality risk in non-periodontitis controls and a substantial frequency in periodontitis patients, we suggest that assessing the CVD mortality risk in a dental setting is worthwhile, especially among periodontitis patients. These findings can contribute to initializing a strategy program for CVD risk detection in a selected at-risk population, as proposed by the European Heart Network (EHN, 2021). These dental profession efforts contribute to the WHO’s goal - to reduce premature death from CVD by 25% by the year 2025 (Sacco et al., 2016).

The European Federation of Periodontology (EFP) commissioned workshop on ‘periodontitis and cardiovascular diseases’ recommended that oral healthcare professionals should assess CVD risk factors in periodontitis patients and inform them accordingly (Sanz et al., 2020a; b). Nevertheless, the implementation of screening for the CVD mortality in the dental setting may be a challenge for many dental practices. Although part of the items to assess the CVD risk are easily available from the patient, such as age, sex, smoking, systolic blood pressure and a self-reported medical history, the challenge may be measuring the blood or plasma biochemical parameters; these biochemical parameters are essential to apply SCORE. Since a large proportion of the population visits the dental practice more often than their general physician, a low-threshold approach to perform blood chemistry procedures during a dental appointment is feasible for dental professionals and patients. Essential blood and plasma markers are accurate and easily obtained with help of the finger stick sampling device method (Huisman, 2017). The maximum duration is 5 minutes and can also be performed by an individual other than an oral healthcare provider, such as a dental nurse. In case of abnormal test results, patients need to be advised to seek diagnostic procedures from a general physician and/or specialist. This will lead to earlier diagnostic procedures at the physicians, enabling patients to start earlier with preventive measures. The implementation of CVD risk assessment in the dental setting should ideally take place in close collaboration with local medical healthcare professionals to provide lifestyle counselling and medication where appropriate.

In conclusion, dentist and periodontist appointments are more frequent and more common than physician and hospital visits before any CVD related complaints or CVD events are apparent. With the current pilot study, we suggest that oral healthcare professionals can play an important role in CVD primary and secondary prevention.

## Data Availability

The raw data supporting the conclusion of this article will be made available by the authors, without undue reservation.
